# Retraction Note: Predictors of sternal wound infection post cardiac surgery in a saudi centre: a case control study

**DOI:** 10.1186/s13019-023-02293-3

**Published:** 2023-05-09

**Authors:** Adil A. Isaac

**Affiliations:** Department of Infectious Diseases, Saud Al-Babtain Cardiac Centre (SBCC), 5443 King Khalid Street, Dammam, Eastern Province Kingdom of Saudi Arabia

**Retraction Note: Journal of Cardiothoracic Surgery (2023) 18:28**

10.1186/s13019-023-02139-y

The Editor-in-Chief has retracted this article as the author had not obtained any ethics approval for this study and did not have permission to publish the data within this article. Adil A. Isaac does not agree to this retraction.

